# The Australian and New Zealand Society for Sarcopenia and Frailty Research (ANZSSFR) sarcopenia diagnosis and management task force: Findings from the consumer expert Delphi process

**DOI:** 10.1111/ajag.13164

**Published:** 2022-12-08

**Authors:** Jesse Zanker, Marc Sim, Kate Anderson, Saliu Balogun, Sharon L. Brennan‐Olsen, Elsa Dent, Gustavo Duque, Christian M. Girgis, Mathis Grossmann, Alan Hayes, Tim Henwood, Vasant Hirani, Charles Inderjeeth, Sandra Iuliano, Justin Keogh, Joshua R. Lewis, Gordon S. Lynch, Julie A. Pasco, Steven Phu, Esmee M. Reijnierse, Nicholas Russell, Lara Vlietstra, Renuka Visvanathan, Troy Walker, Debra L. Waters, Solomon Yu, Andrea B. Maier, Robin M. Daly, David Scott

**Affiliations:** ^1^ Australian Institute for Musculoskeletal Science (AIMSS) The University of Melbourne and Western Health Melbourne Victoria Australia; ^2^ Department of Medicine – Western Health The University of Melbourne Melbourne Victoria Australia; ^3^ Nutrition and Health Innovation Research Institute, School of Medical and Health Sciences Edith Cowan University Joondalup Western Australia Australia; ^4^ School of Medicine University of Western Australia Perth Western Australia Australia; ^5^ Institute for Health Transformation – Determinants of Health, Faculty of Health Deakin University Melbourne Victoria Australia; ^6^ School of Health and Social Development, Faculty of Health Deakin University Melbourne Victoria Australia; ^7^ College of Health and Medicine Australian National University Canberra Australian Capital Territory Australia; ^8^ Menzies Institute for Medical Research University of Tasmania Hobart Tasmania Australia; ^9^ Institute for Health Transformation Deakin University Melbourne Victoria Australia; ^10^ Torrens University Australia Adelaide South Australia Australia; ^11^ Department of Medicine Research Institute of the McGill University Health Centre McGill University Montreal Quebec Canada; ^12^ Faculty of Medicine and Health University of Sydney Sydney New South Wales Australia; ^13^ Department of Diabetes and Endocrinology Westmead Hospital Sydney New South Wales Australia; ^14^ Department of Medicine – Austin Health The University of Melbourne Melbourne Victoria Australia; ^15^ Department of Endocrinology Austin Health Melbourne Victoria Australia; ^16^ Institute for Health and Sport (IHeS) Victoria University Melbourne Victoria Australia; ^17^ Human Movement and Nutritional Science University of Queensland Brisbane Queensland Australia; ^18^ Nutrition and Dietetics Group, School of Life and Environmental Sciences Charles Perkins Centre University of Sydney Sydney New South Wales Australia; ^19^ North Metropolitan Health Service and University of Western Australia Perth Western Australia Australia; ^20^ Faculty of Health Sciences and Medicine Bond University Gold Coast Queensland Australia; ^21^ Human Potential Centre Auckland University of Technology Auckland New Zealand; ^22^ Cluster for Health Improvement, Faculty of Science, Health, Education and Engineering University of the Sunshine Coast Sunshine Coast Queensland Australia; ^23^ Kasturba Medical College, Mangalore Manipal Academy of Higher Education Manipal Karnataka India; ^24^ Centre for Kidney Research, Children's Hospital at Westmead School of Public Health, Sydney Medical School The University of Sydney Sydney Australia; ^25^ Centre for Muscle Research, Department of Anatomy and Physiology, School of Biomedical Sciences The University of Melbourne Melbourne Victoria Australia; ^26^ IMPACT‐Institute for Mental and Physical Health and Clinical Translation, Barwon Health Deakin University Melbourne Victoria Australia; ^27^ Falls, Balance, and Injury Research Centre Neuroscience Research Australia (NeuRA) Sydney New South Wales Australia; ^28^ Department of Medicine and Aged Care, The Royal Melbourne Hospital The University of Melbourne Melbourne Victoria Australia; ^29^ Amsterdam UMC, Vrije Universiteit Amsterdam, Rehabilitation Medicine Amsterdam The Netherlands; ^30^ Amsterdam Movement Sciences, Ageing and Vitality Amsterdam The Netherlands; ^31^ School of Physical Education, Sport and Exercise Sciences University of Otago Dunedin New Zealand; ^32^ Adelaide Geriatrics Training and Research with Aged Care (GTRAC) Centre, School of Medicine, Faculty of Health and Medical Sciences University of Adelaide Adelaide South Australia Australia; ^33^ Aged and Extended Care Services, Acute and Urgent Care, The Queen Elizabeth Hospital, Central Adelaide Local Health Network Adelaide South Australia Australia; ^34^ Institute for Health Transformation, Global Obesity Centre Deakin University Melbourne Victoria Australia; ^35^ Department of Medicine, School of Physiotherapy University of Otago Dunedin New Zealand; ^36^ Healthy Longevity Translational Research Program, Yong Loo Lin School of Medicine National University of Singapore Singapore; ^37^ Centre for Healthy Longevity National University Health System Singapore; ^38^ Department of Human Movement Sciences, Faculty of Behavioural and Movement Sciences Vrije Universiteit Amsterdam Amsterdam The Netherlands; ^39^ Institute for Physical Activity and Nutrition Deakin University Melbourne Victoria Australia; ^40^ Department of Medicine, School of Clinical Sciences at Monash Health Monash University Melbourne Victoria Australia

**Keywords:** community‐based participatory research, geriatric assessment, sarcopenia

## Abstract

**Objectives:**

To develop guidelines, informed by health‐care consumer values and preferences, for sarcopenia prevention, assessment and management for use by clinicians and researchers in Australia and New Zealand.

**Methods:**

A three‐phase Consumer Expert Delphi process was undertaken between July 2020 and August 2021. Consumer experts included adults with lived experience of sarcopenia or health‐care utilisation. Phase 1 involved a structured meeting of the Australian and New Zealand Society for Sarcopenia and Frailty Research (ANZSSFR) Sarcopenia Diagnosis and Management Task Force and consumer representatives from which the Phase 2 survey was developed. In Phase 2, consumers from Australia and New Zealand were surveyed online with opinions sought on sarcopenia outcome priorities, consultation preferences and interventions. Findings were confirmed and disseminated in Phase 3. Descriptive statistical analyses were performed.

**Results:**

Twenty‐four consumers (mean ± standard deviation age 67.5 ± 12.8 years, 18 women) participated in Phase 2. Ten (42%) identified as being interested in sarcopenia, 7 (29%) were health‐care consumers and 6 (25%) self‐reported having/believing they have sarcopenia. Consumers identified physical performance, living circumstances, morale, quality of life and social connectedness as the most important outcomes related to sarcopenia. Consumers either had no preference (46%) or preferred their doctor (40%) to diagnose sarcopenia and preferred to undergo assessments at least yearly (54%). For prevention and treatment, 46% of consumers preferred resistance exercise, 2–3 times per week (54%).

**Conclusions:**

Consumer preferences reported in this study can inform the implementation of sarcopenia guidelines into clinical practice at local, state and national levels across Australia and New Zealand.

**Practice Impact**

This Consumer Expert Delphi study captured the experience of health‐care consumers and older adults living with sarcopenia to inform clinical and research recommendations made by the ANZSSFR. We present a framework for the engagement of consumers in the development of clinical guidelines.

## 1 INTRODUCTION

Person‐centred research, which focuses on the opinions of health‐care consumers and those with lived experience of a medical condition (*consumer experts*), is increasingly important for quality health‐care delivery.^1^ Older European adults with lived experience of sarcopenia, a highly prevalent condition of progressive and accelerated loss of muscle strength, mass and physical performance,^2^ report that the sarcopenia‐related outcomes most important to them are difficulties with mobility and domestic activities, falls, fatigue and quality of life.^3^, ^4^

Outcomes of importance to health‐care consumers in Australia and New Zealand, and their preferences regarding the assessment and management of sarcopenia, are unclear. Building on our recent collaborative work,^5^ the Australian and New Zealand Society for Sarcopenia and Frailty Research (ANZSSFR) Task Force for Sarcopenia Diagnosis and Management (herein Task Force) sought to answer these questions by facilitating a modified Consumer Expert Delphi study in parallel with a Topic Expert Delphi study.^6^

To ensure that consumer input informs the development of person‐centred sarcopenia guidelines in Australia and New Zealand,^6^ the aim of this study was to determine the opinions and preferences of consumer experts on sarcopenia outcomes and practice.

## 2 METHODS

This three‐phase modified consumer Delphi method was undertaken between July 2020 and August 2021. The Delphi method has been recommended for person‐centred research^1^ and is a process of structured communication that supports a group of individuals to collectively address complex problems and reach a consensus.^7^ We adhered to standard procedures for a Delphi study.^8^ This study was approved by Monash Health Human Research Ethics Committee (ERM 64175).

### 2.1 Participants and survey development

Task Force members (*n* = 29) participated in Phases 1 and 3. Task Force recruitment is described in both previous and parallel *Topic Expert Delphi* studies.^5^, ^6^ Phase 1 of this *Consumer Expert Delphi* involved a videoconference to collaboratively develop questions among the Task Force. Three consumer representatives associated with Task Force member institutions provided feedback on survey content and design. The survey included discrete choice experiment (DCE) questions^4^ with Likert ranking scales and personal preference questions on outcomes related to, and assessment and management of, sarcopenia. Participants could provide free‐text responses to each question.

**FIGURE 1:**
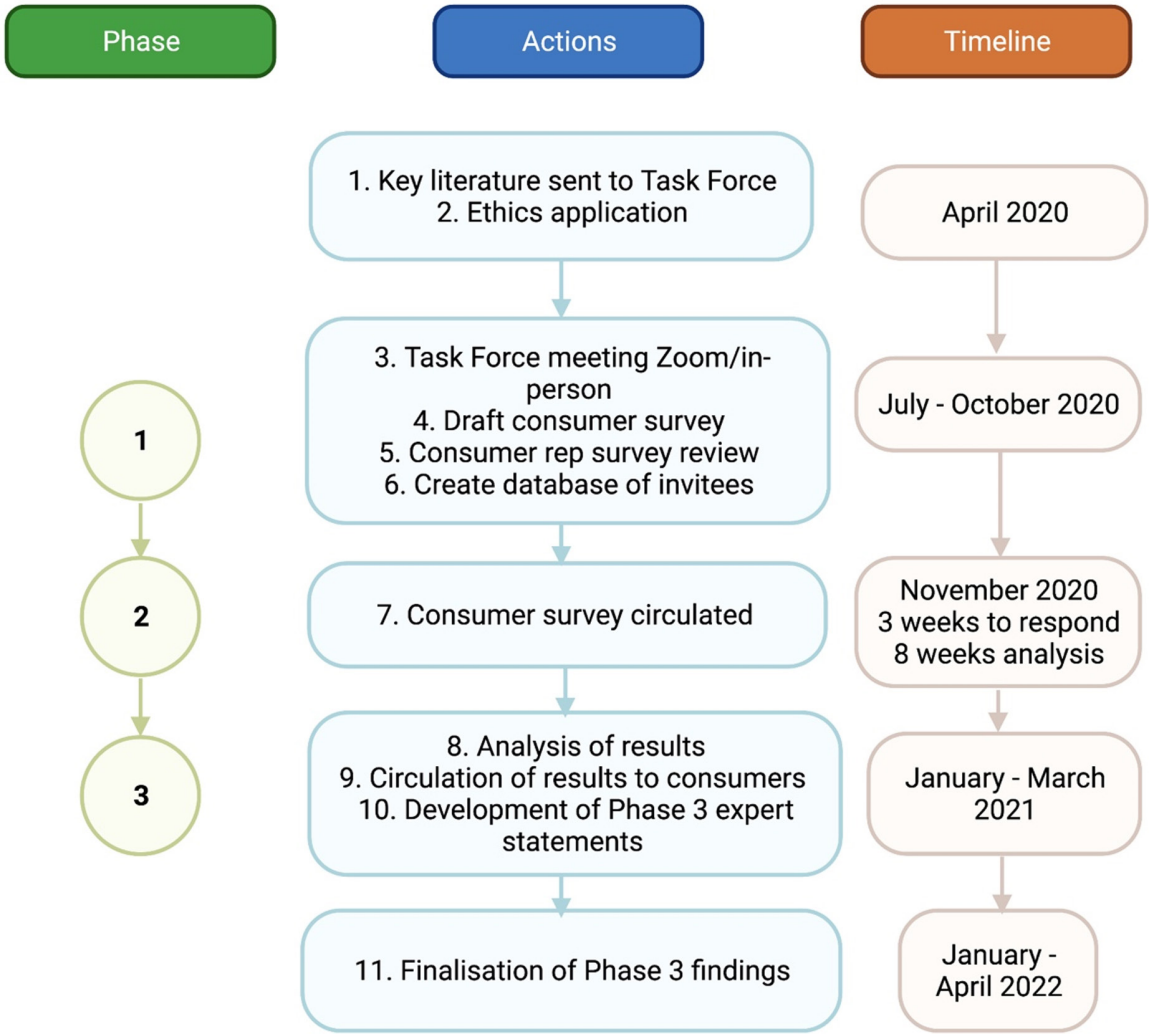
Flow chart of consumer Delphi study

Email invitations were sent to consumer groups across Australia and New Zealand, and Task Force members used existing networks and social media (e.g. LinkedIn) to invite consumer participants. A flow diagram is presented in Figure 1. All participants provided written informed consent via email (Appendix S1). An online survey (Qualtrics) was completed anonymously by consumer experts in Phase 2 (Appendix S2). Results of Phase 2 were provided to participating consumers via an accessible infographic (Appendix S3). The finalisation of results for dissemination was completed by the Task Force in Phase 3.

### 2.2 Analysis

The Delphi method is an iterative process, thus Phases 2 and 3 were informed by findings from prior phases. Descriptive statistical analyses, and critical analyses of free text, were performed. Phase 2 *Consumer Expert Delphi* findings influenced the content and presentation of the parallel *Topic Expert Delphi* findings.^6^

## 3 RESULTS

This three‐phase modified *Consumer Expert Delphi* study involved health‐care consumers (Phase 1, *n* = 3; Phase 2, *n* = 24) and Task Force experts (Phases 1 and 3, *n* = 29). The demographic details of Task Force experts are described elsewhere.^6^ All 24 consumer survey responses were valid and included. Consumers participating in Phase 2 (*n* = 24), had mean ± SD age of 67.5 ± 12.8 years (Table 1) and 16 (67%) were women. The majority (*n* = 10, 42%) described themselves as ‘interested in sarcopenia’, followed by ‘consumer of healthcare’, (*n* = 7, 29%). Six (25%) people were ‘living with sarcopenia’ or ‘believed they had sarcopenia’ and one (4%) described themselves as a ‘carer’. The majority (*n* = 18, 75%) were from Victoria with two consumer experts (8%) from Tasmania, Western Australia and New Zealand each. Most participants were self‐funded retirees (*n* = 11, 46%) and one quarter (*n* = 6, 25%) were on government support pensions or in paid employment.

**TABLE 1:** Phase 2 consumer demographics

Characteristic	Sub‐category	*n* = 24
Mean age, years (SD)		67.5 (13)
Gender, *n* (woman, %)		16 (67)
Self‐description, *n* (%)	Person interested in sarcopenia	10 (42)
	Consumer of health‐care services	7 (29)
	Person who believes that he/she is, or maybe at risk of, living with sarcopenia	4 (17)
	Person living with sarcopenia	2 (8)
	Carer for someone with sarcopenia	1 (4)
Location, *n* (%)	Victoria	18 (75)
	Tasmania	2 (8)
	Western Australia	2 (8)
	New Zealand	2 (8)
Background and descent, *n* (%)	European/Caucasian	23 (96)
	Asian	1 (4)
First language, *n* (%)	English	24 (100)
Language spoken at home, *n* (%)	English	24 (100)
Main income source, *n* (%)	Self‐funded retiree	11 (46)
	Government support pension (aged/disability, etc.)	6 (25)
	Paid employment	6 (25)
	Other (not specified)	1 (4)

Abbreviation: SD, standard deviation.

In Phase 2, consumer experts were asked to rate the importance (from not important = 0 to very important = 10) of 23 clinical outcomes of sarcopenia. Very important outcomes (>80% of consumers rated as 8, 9 or 10) included lower physical function, reduced muscle strength, reduced mobility, fatigue, loss of balance, falls (including fear of falling), fractures, risk of hospitalisation, loss of independence, institutionalisation, mood problems, lower morale, lower mental function, social isolation and reduced quality of life. Increased risk of falls (*n* = 24, 100%) and loss of balance (*n* = 22, 92%) were ranked as most important, whereas outcomes identified as least important were altered physical appearance and difficulty with tasks at home (both *n* = 16, 67%).

### 3.1 Sarcopenia diagnosis and management

Consumer experts provided preferences on the assessment and management of sarcopenia, and willingness to engage in research (Appendix S4). Regarding assessment, consumers mostly reported they ‘don't mind who diagnoses sarcopenia’ (*n* = 11, 46%), and the majority would be willing to undertake all assessments and tests deemed necessary to make the diagnosis (*n* = 21, 88%). Consumers reported they would prefer consultation lengths of greater than 60 minutes (or the required duration needed to make an assessment; *n* = 10, 42%), followed by 30–60 min (*n* = 9, 38%). Consumers mostly preferred consultation frequency as recommended by their health‐care professional (*n* = 9, 38%); although combined, the majority of consumers (*n* = 13, 54%) would be willing to undertake a consultation every 6 months or less (*n* = 7, 29%) or yearly (*n* = 6, 25%).

### 3.2 Sarcopenia prevention

For sarcopenia prevention, consumers mostly preferred to undertake resistance exercise (*n* = 18, 75%) followed by taking prescription medications (if available; *n* = 17, 71%) and making dietary modifications (*n* = 16, 67%). The single most preferred activity to prevent sarcopenia was resistance exercise (*n* = 11, 46%), with a majority identifying the optimal frequency as 2–3 times per week (*n* = 13, 54%).

### 3.3 Sarcopenia treatment

To treat sarcopenia, exercise frequency of 2–3 times per week was most preferred by consumers (*n* = 11, 46%). Most consumers identified willingness to be involved in research for both exercise (*n* = 21, 88%) and dietary (*n* = 15, 62%) studies. However, only one‐third (*n* = 8, 33%) of consumers expressed willingness to be involved in clinical trials of medication for sarcopenia.

### 3.4 Phase 3 finalisation

Twenty‐nine Task Force members reviewed Phase 2 findings, confirmed interpretation and impact on Phase 3 survey of the parallel *Topic Expert Delphi*.^6^

## 4 DISCUSSION

The findings of our *Consumer Expert Delphi* study, demonstrating sarcopenia outcome priorities for health‐care consumers (e.g., avoiding falls) and treatment preferences (e.g., resistance exercise), contrasted some of the findings of, and influenced multiple statements contained within, our parallel *Topic Expert Delphi* study.^6^ This suggests that consumers' values and preferences with regard to health care may differ from those of experts, with such differences needing to be incorporated into health‐care guidelines.

Lived experience and sarcopenia research to date has examined consumer priorities regarding outcomes but not health‐care preferences.^3^, ^4^ No study to our knowledge has obtained consumer preferences on sarcopenia assessment, prevention, treatment and research, and combined these findings to inform contemporaneous clinical guidelines that can be implemented as a best practice, person‐centred care.

Both consumer and topic experts agreed that falls, mobility and quality of life were important outcomes of sarcopenia.^5^ Our findings are consistent with studies of older European adults living with sarcopenia who prioritised outcomes including mobility, falls, fatigue and quality of life identified as most important.^3^, ^4^ However, 91% of respondents in the *Topic Expert Delphi* reported that functional status (i.e. the ability to undertake activities of daily living) was important as compared with the *Consumer Expert Delphi* finding that one's ‘ability to undertake tasks around the home’ was of least importance.^6^ Interestingly, this finding also contrasted with European consumer studies, where ‘management of domestic duties’ was identified as the second most important outcome.^3^, ^4^ The difference in importance of management of domestic duties for these populations may relate to underlying roles within households reflecting ethnographic, gender and study population differences.^3^, ^4^ Regardless, this finding suggests that older adults in Australia and New Zealand may prioritise outcomes differently, and management approaches must consider individual values and preferences.

Consumer experts identified resistance exercise as the action they were most willing to undertake to prevent and treat sarcopenia, followed by prescription medications and dietary modifications. Resistance exercise is indeed the primary recommended therapy for the prevention and treatment of sarcopenia,^9^, ^10^ suggesting a high degree of health literacy in this population of consumers, a high proportion of whom were self‐funded retirees or currently employed. The clinical implications of these findings depend upon the uptake and implementation of the guidelines this study informed,^6^ but critically, clinician interventions when following these clinical guidelines will be embedded with the preferences of consumer experts. This may better support consumers to actively participate in decision‐making and planning for their health care for sarcopenia, and contribute to greater engagement and adherence to sarcopenia case finding and interventions.

### 4.1 Limitations

Our study was limited by its small, targeted sample size and low representation of people living with sarcopenia. This may reflect relatively low public awareness of sarcopenia^11^ and highlights the need for educational campaigns to improve the understanding of sarcopenia in Australia and New Zealand. This is exacerbated by the lack of a local consensus definition of sarcopenia until recently.^6^ Future consumer‐focused Delphi processes should concentrate on increasing the engagement of those at risk or living with sarcopenia. Second, our study's diversity was limited by the participant demographics (e.g. a high proportion were Caucasian), which are not representative of older adult population in Australia and New Zealand, thus affecting generalisability. Third, response bias may have influenced results as consumer experts answered theoretical questions and were not faced with real decisions about their health care.^12^ However, the findings were nonetheless valuable in informing the *Topic Expert Delphi* in line with best practice^1^ and led to the modification of clinical guidelines reflecting consumer values and preferences.

## 5 CONCLUSIONS

This novel, modified *Consumer Expert Delphi* study of Australian and New Zealand older adults found that perspectives of sarcopenia outcomes, assessment and management may differ between health‐care consumers and field experts, particularly with reference to outcome priorities. Our study findings contribute to and highlight the importance of person‐centred consumer input in the development of clinical guidelines.

## CONFLICTS OF INTEREST

ABM has received speaker and consulting fees from Abbott, Nutricia, AstraZeneca and Novartis. GD is a member of the Scientific Advisory Board of TSI, Abbott and Amgen and has received speaker/consulting fees from Amgen, Abbott and TSI. MG has received research funding from Bayer Pharma, Novartis, Weight Watchers, Lilly, Otsuka and speaker's honoraria from Bayer Pharma, Besins Healthcare and Amgen. RMD reports a grant from Fonterra Co‐operative Group Ltd, honoraria for presentations from Abbott Australia and Nutricia Research and to serve as a member of an expert advisory committee. LV is an Associate Editor of the Australasian Journal on Ageing. RV has previously received education and honoraria from the following Abbott, Nestlé and Nutricia. DLW is Editor‐in‐Chief of the Australasian Journal on Ageing. SI has received speaker/consulting fees from Abbott, UK Dairy Council, European Milk Forum, Nestlé Health Science and the Israel Milk Board.

## Supporting information


Appendix S1

Appendix S2

Appendix S3

Appendix S4

## Data Availability

Data are available on request due to privacy/ethical restrictions.
