# Supplementary material for: The Australian and New Zealand Society for Sarcopenia and Frailty Research (ANZSSFR) sarcopenia diagnosis and management task force: Findings from the consumer expert Delphi process
Source: Australas J Ageing. 2022 Dec 8;42(1):251–7. doi: 10.1111/ajag.13164 (PMC10947359; doi:10.1111/ajag.13164)
Supplement: Supplementary file 1 — Appendix S1 [file AJAG-42-251-s004.docx]

**Appendix 1 – Participant informed consent form**

**The Australian and New Zealand Society for Sarcopenia and Frailty Research Task Force on Sarcopenia Diagnosis and Management**

**Modified Delphi Study**

**Non-Task Force Participant
Participant Information and Consent Form**

**Plain Language Summary**

Sarcopenia is a condition which results in loss of muscle strength, mass, and physical performance (such as walking speed). Sarcopenia is common among older adults, particularly those with multiple medical problems or those living in residential care. It can also be related to a sedentary lifestyle and lack of physical activity. Sarcopenia can contribute to a range of problems, including falls, fractures and death. Despite being an important condition, there is no agreed way to define sarcopenia. There are no clear guidelines for health professionals to treat sarcopenia in Australia and New Zealand. The effects of sarcopenia that are actually important to people with the condition, such as falls or difficulty with household tasks, are unknown. It is also not known what sort of health assessment and treatment people with sarcopenia would be willing to undertake or what options are available to them. In this study, we will seek the opinions of a range of people including sarcopenia experts, health professionals, community members, people with sarcopenia and the caregivers of people living with sarcopenia to produce recommendations for researchers and clinicians in Australia and New Zealand working with people living with or at risk of sarcopenia.

**Consent**

You have expressed interest in participating in two anonymous, online surveys. You will be asked to provide your opinion on a number of statements relating to sarcopenia. You will be asked some demographic questions, but you will have the opportunity to select ‘prefer not to say’. You will have the opportunity to withdraw your involvement at any stage of the study. You will not be asked any identifying or personal/health-related questions and your responses will be confidential and unable to be linked to you.

After providing your email address, you will be contacted with an additional survey link when the survey is open.

**Please read the ‘explanatory statement’ for more detailed information.**

**Click on the link below that applies to you. This will allow you to send an email which gives your consent to be sent a survey link. Be sure to click ‘send’ on the email. You do not have to write anything in the body of the email.**

[If you are a](mailto:david.scott@monash.edu?subject=Consumer%20-%20I%20consent%20to%20my%20involvement%20in%20the%20ANZSSFR%20Task%20Force%20Delphi%20Study) **[community member](mailto:david.scott@monash.edu?subject=Consumer%20-%20I%20consent%20to%20my%20involvement%20in%20the%20ANZSSFR%20Task%20Force%20Delphi%20Study)**[, carer](mailto:david.scott@monash.edu?subject=Consumer%20-%20I%20consent%20to%20my%20involvement%20in%20the%20ANZSSFR%20Task%20Force%20Delphi%20Study) **[for someone with or at risk of sarcopenia](mailto:david.scott@monash.edu?subject=Consumer%20-%20I%20consent%20to%20my%20involvement%20in%20the%20ANZSSFR%20Task%20Force%20Delphi%20Study)**[, or a person with](mailto:david.scott@monash.edu?subject=Consumer%20-%20I%20consent%20to%20my%20involvement%20in%20the%20ANZSSFR%20Task%20Force%20Delphi%20Study) **[or at risk for sarcopenia](mailto:david.scott@monash.edu?subject=Consumer%20-%20I%20consent%20to%20my%20involvement%20in%20the%20ANZSSFR%20Task%20Force%20Delphi%20Study)**[:](mailto:david.scott@monash.edu?subject=Consumer%20-%20I%20consent%20to%20my%20involvement%20in%20the%20ANZSSFR%20Task%20Force%20Delphi%20Study)

[Click here to provide your email address and consent to your involvement in the study.](mailto:david.scott@monash.edu?subject=Consumer%20-%20I%20consent%20to%20my%20involvement%20in%20the%20ANZSSFR%20Task%20Force%20Delphi%20Study)

[Click here to provide your email address and consent to your involvement in the study](mailto:david.scott@monash.edu?subject=Consumer%20-%20I%20consent%20to%20my%20involvement%20in%20the%20ANZSSFR%20Task%20Force%20Delphi%20Study).

OR

[If you are a](mailto:david.scott@monash.edu?subject=Expert%20-%20I%20consent%20to%20my%20involvement%20in%20the%20ANZSSFR%20Task%20Force%20Delphi%20Study) **[health professional, or a researcher with expertise on sarcopenia:](mailto:david.scott@monash.edu?subject=Expert%20-%20I%20consent%20to%20my%20involvement%20in%20the%20ANZSSFR%20Task%20Force%20Delphi%20Study)**

[Click here to provide your email address and consent to your involvement in the study.](mailto:david.scott@monash.edu?subject=Expert%20-%20I%20consent%20to%20my%20involvement%20in%20the%20ANZSSFR%20Task%20Force%20Delphi%20Study)

**EXPLANATORY STATEMENT – NON-TASK FORCE PARTICIPANT**

**This information sheet is for you to keep**

**Invitation to Participate:** You have expressed an interest in participating in a research survey study to develop recommendations on the diagnosis and management of sarcopenia in Australia and New Zealand. Please read this Explanatory Statement in full before deciding whether or not to participate in this research. If you would like further information regarding any aspect of this project, you are encouraged to contact the researchers via the phone number or email address listed above.

**Purpose of the research:** The Australian and New Zealand Society for Sarcopenia and Frailty Research (the *Society*) is a not-for-profit organisation committed to improving knowledge and translation of research on sarcopenia and frailty. In 2018, the *Society* established a Task Force whose objective was to establish a definition of sarcopenia in Australia and New Zealand. The Task Force now seeks the opinions of a wide-range of stakeholders in Australia and New Zealand to determine preferred recommendations on the diagnosis and management of sarcopenia. These recommendations will be formed by a modified Delphi process, a method of surveying large groups of people to achieve consensus.

**What the research involves:** You have been invited to participate because you have indicated your interest in, or are involved in research or clinical care of older adults who may experience sarcopenia.

Should you choose to participate in this study, you will be invited to:

- Complete two online, anonymous surveys
- The surveys will take place over two weeks in November 2020 and February 2021

**Funding**: This project is being supported in-kind through website advertising and survey software by the *Society*.

**Consenting to participate in the project and withdrawing from the research:** Your participation in the research study will start when you have read this Explanatory Statement and clicked the link to provide your email address. Participation in this research project is voluntary. If you do not wish to take part, you do not have to. If you decide to take part and later change your mind, you are free to withdraw from the project. However, you will only be able to withdraw your responses prior to completion of the survey. At the end of the online survey, you will be notified the survey has come to an end. Throughout the survey, you can ‘go back’ to revise your answers. After you submit the survey and are notified at the end, any data you provide will be unidentifiable.

**Implications of declining participation or withdrawal:** Your decision to participate or not participate, as well as your decision to withdraw from the study at a later date, will have NO impact on you. You will not be contacted to provide any explanation for declining to participate or withdrawing.

**Remuneration:** There is no remuneration for participation in this project.

**Possible benefits and risks to participants:** You may experience minor inconvenience or discomfort relating to the time taken to participate in this study or confronting information about possible outcomes linked to sarcopenia. There is no immediate personal benefit from participation in this study. However, your participation will contribute to the recommendations presented by the *Society* Task Force.

**Services on offer if adversely affected:** In the unlikely event that you experience distress during or after participation is this study, please contact, free telephone-based counselling services. This service is not affiliated with the researchers.
Beyondblue 1300 22 4636
Lifeline 13 11 14

**Confidentiality:** Your participation and any information you provide will be kept confidential. You will be asked some demographic questions, but you will have the opportunity to select, ‘prefer not to say.’ You will have the opportunity to withdraw your involvement at any stage of the study. You will not be asked any identifying or personal/health-related questions and your responses will be confidential and unable to be linked to you. Responses will be reported as a summary of group results in such a way that no one can be identified.

**Storage of data:** Anonymous, non-identifiable data will be stored in a password-protected file on a network drive accessible only using the Principal Investigator's computer at the University of Melbourne. Email addresses will be stored in a password-protected Access file stored on a secure server and no other identifying information will be included in this file. The files will be stored on the secure network and backed up daily. No hard copy documents will be collected. Data will be stored for a period of fifteen years after the publication of results. After this, data will be destroyed in an appropriate manner. It is not possible for the data collected to be used for other purposes such as conversion to health information.

**Results:** The results of each phase will be shared with all participants. A scientific manuscript will be prepared for submission to a high impact journal following Task Force approval. Submissions will also be made to a conference deemed appropriate by the Task Force.

**Is this research project approved?**

This project will be carried out according to the *National Statement on Ethical Conduct in Human Research (2018)* produced by the National Health and Medical Research Council of Australia. This statement has been developed to protect the interests of people who agree to participate in human research studies.

The ethical aspects of this research project have been approved by the Monash Health Human Research Ethics Committee (ref: ERM 64175).
