# Supplementary material for: The Australian and New Zealand Society for Sarcopenia and Frailty Research (ANZSSFR) sarcopenia diagnosis and management task force: Findings from the consumer expert Delphi process
Source: Australas J Ageing. 2022 Dec 8;42(1):251–7. doi: 10.1111/ajag.13164 (PMC10947359; doi:10.1111/ajag.13164)
Supplement: Supplementary file 2 — Appendix S2 [file AJAG-42-251-s001.docx]

**Appendix 2 – Phase 2 Consumer survey**

**Australian and New Zealand Society for Sarcopenia and Frailty Research Sarcopenia Diagnosis and Management Task Force (ANZSSFR) Sarcopenia Diagnosis and Management Task Force**

**Phase 2 Consumer Survey**

**Page 1**

**Plain Language Summary**

Sarcopenia is a condition characterised by loss of muscle mass and strength. This results in reduced physical performance, such as slow walking speed. Sarcopenia is common among older adults, particularly those with multiple medical problems or those living in residential care. Sarcopenia can also be related to a sedentary lifestyle and lack of physical activity. Sarcopenia can contribute to a range of problems, including falls, fractures and death. Despite being an important condition, there is no agreed way to define sarcopenia. There are no clear guidelines for health professionals to treat sarcopenia in Australia and New Zealand. The effects of sarcopenia that are actually important to people with the condition, such as falls or difficulty with household tasks, are unknown. It is also not known what sort of health assessment and treatment people with sarcopenia would be willing to undertake or what options are available to them. In this study, we will seek the opinions of a range of people; including sarcopenia experts, health professionals, community members, people with sarcopenia and their caregivers. With this we aim to produce recommendations for the researchers and clinicians in Australia and New Zealand who work with people who live with or are at risk of sarcopenia.

**Page 2**

Thank you for your active participation in this important study on sarcopenia in Australia and New Zealand.

Please refer to our *Explanatory Statement* for more information. This is available on the ANZSSFR website at<https://anzssfr.org/> or can be sent to you via email. Email [dscott@monash.edu](mailto:dscott@monash.edu) to receive a copy of the explanatory statement.

Through this multi-stage process of consulting key stakeholders, such as yourself, we aim to establish consensus recommendations for the diagnosis and management of sarcopenia in Australia and New Zealand. This process is called a ‘Delphi Method’. Your participation in this survey, and a shorter follow-up survey, will greatly assist in achieving and promoting these consensus recommendations. We are most grateful for your valuable contribution.

You have consented to involvement in Phases 2 and 3 of the Delphi Method, which are short online surveys, where you are asked for your opinion on a range of questions and statements. Each survey will take less than 15 minutes. The statements presented are informed by research, but we are interested in your thoughts about these statements and questions. These readings are not compulsory for you to respond to the Phases 2 and 3 of the Delphi questions and statements.

**Page 3**

Thank you for agreeing to participate in our study. All viewpoints are valued and it is important that you complete the questionnaires in both Phase 2 and Phase 3.

Your participation is voluntary and anonymous. Thank you for your valuable contribution.

**Completing the survey**

You will be asked questions about yourself and then on your opinions about sarcopenia. For some questions you will be asked to choose options, for others, you will be asked to rank which statement best describes your opinion, and so on. You can select, ‘I don’t have an opinion on this’ or leave the answer blank if you are unsure of the content or how to answer. You can add extra comments with each question if you wish.

**Page 4**

*About you – consumer/carer details*

Which best describes you? Person living with sarcopenia / person who believes they are, or may be at risk of, living with sarcopenia / person interested in sarcopenia (please specify e.g., for general knowledge, personal well-being etc.) /carer for someone with sarcopenia / consumer of health care services

Age [insert whole number]

Gender M/F/Other (please specify) / Prefer not to say

State/Country Drop-down list / prefer not to say

Do you identify as Aboriginal/Torres Strait Islander/Māori/Pacific Islander/Asian descent/Caucasian/European descent/African descent/South American descent/Other (please specify)
- You can select more than one
Prefer not to say

Which is your first language? English/Other – please specify

Which language do you Free text
speak at home?

What is your main income Paid employment/carers support/pension (aged/disability) self-funded

**Page 5**

**Sarcopenia outcomes**

1. Sarcopenia has been linked with a range of different consequences, known as ‘outcomes’.

Please give each ‘outcome’ a number depending on how important you think it is for people to avoid these outcomes. ‘1’ means ‘not important at all to avoid the outcome’ and ‘10’ means ‘very important to avoid the outcome’.

| **Outcome** | **Not important Very important** | | | | | | | | | |
| --- | --- | --- | --- | --- | --- | --- | --- | --- | --- | --- |
| Lower physical function | 1 | 2 | 3 | 4 | 5 | 6 | 7 | 8 | 9 | 10 |
| Reduced muscle strength | 1 | 2 | 3 | 4 | 5 | 6 | 7 | 8 | 9 | 10 |
| Reduced mobility (e.g., unable to walk around the home or community) | 1 | 2 | 3 | 4 | 5 | 6 | 7 | 8 | 9 | 10 |
| Slower walking speed | 1 | 2 | 3 | 4 | 5 | 6 | 7 | 8 | 9 | 10 |
| Difficulty with personal tasks (showering, toileting etc.) | 1 | 2 | 3 | 4 | 5 | 6 | 7 | 8 | 9 | 10 |
| Difficulty with tasks at home (cleaning, cooking etc.) | 1 | 2 | 3 | 4 | 5 | 6 | 7 | 8 | 9 | 10 |
| Difficulty carrying moderate to heavy loads (e.g., grocery bags) | 1 | 2 | 3 | 4 | 5 | 6 | 7 | 8 | 9 | 10 |
| Fatigue | 1 | 2 | 3 | 4 | 5 | 6 | 7 | 8 | 9 | 10 |
| Loss of appetite | 1 | 2 | 3 | 4 | 5 | 6 | 7 | 8 | 9 | 10 |
| Loss of balance | 1 | 2 | 3 | 4 | 5 | 6 | 7 | 8 | 9 | 10 |
| Increased risk of falls | 1 | 2 | 3 | 4 | 5 | 6 | 7 | 8 | 9 | 10 |
| Fractures (broken bones) | 1 | 2 | 3 | 4 | 5 | 6 | 7 | 8 | 9 | 10 |
| Increased risk of hospitalisation | 1 | 2 | 3 | 4 | 5 | 6 | 7 | 8 | 9 | 10 |
| Not being able to live in one’s own home | 1 | 2 | 3 | 4 | 5 | 6 | 7 | 8 | 9 | 10 |
| Needing to move into a Residential aged care facility | 1 | 2 | 3 | 4 | 5 | 6 | 7 | 8 | 9 | 10 |
| Altered physical appearance (less muscle, looking ‘frail’) | 1 | 2 | 3 | 4 | 5 | 6 | 7 | 8 | 9 | 10 |
| Mood problems, such as depression or anxiety | 1 | 2 | 3 | 4 | 5 | 6 | 7 | 8 | 9 | 10 |
| Lower morale | 1 | 2 | 3 | 4 | 5 | 6 | 7 | 8 | 9 | 10 |
| Lower mental function | 1 | 2 | 3 | 4 | 5 | 6 | 7 | 8 | 9 | 10 |
| Fear of falls | 1 | 2 | 3 | 4 | 5 | 6 | 7 | 8 | 9 | 10 |
| Social isolation (e.g., unable to visit friends/family) | 1 | 2 | 3 | 4 | 5 | 6 | 7 | 8 | 9 | 10 |
| Poor quality of life | 1 | 2 | 3 | 4 | 5 | 6 | 7 | 8 | 9 | 10 |
| Increased risk of death | 1 | 2 | 3 | 4 | 5 | 6 | 7 | 8 | 9 | 10 |

Please comment if you wish __________________________________________________

**Assessment of sarcopenia**

The following questions ask you to think about the assessments and tests you might be requested to undertake if you were to be assessed for sarcopenia.

1. Please select the statement which best reflects your opinion:

- I would prefer that my GP diagnose sarcopenia
- I would prefer that an allied health professional (such as a physiotherapist, exercise physiologist, chiropractor, osteopath or dietitian) diagnose sarcopenia
- I would prefer that a specialist (e.g., geriatrician) diagnose sarcopenia
- I don’t mind who diagnoses sarcopenia
- I do not have an opinion on this

Please comment if you wish __________________________________________________

An ‘assessment’ is a measure of strength or something physical. To assess a person for sarcopenia, sometimes the healthcare professional may request that you undertake physical assessments, such as measuring your hand grip strength or the speed you can walk over a short distance, such as 4 metres.

Further tests may also be recommended. A ‘test’ means a procedure involving a machine, such as an X-ray. Tests may include a type of X-ray called DXA (pronounced ‘dexa’), which can be used to measure the amount of muscle and fat in the body. DXA tests have much lower radiation than a standard X-ray. ‘Radiation’ describes the effect the X-ray can have on the body, which can sometimes be harmful.

Other tests, such as a BIA, is a set of scales that you stand on and hold two handles. It measures water, fat and everything that is not fat or bone in the body. There is no radiation in a BIA test but it may not be as precise as DXA.

1. Please select the statement which best reflects your opinion:

- I would be willing to undertake all the necessary ASSESSMENTS and TESTS to diagnose sarcopenia
- I would only be willing to undertake one or two ASSESSMENTS to diagnose sarcopenia
- I would only be willing to undertake one or two TESTS to diagnose sarcopenia
- I would prefer to not undertake TESTS, but would be happy with ASSESSMENTS
- I would prefer not to undertake ASSESSMENTS or TESTS
- I do not have an opinion on this

Please comment if you wish __________________________________________________

1. The following question refers to the length of a consultation that would be acceptable to you when being assessed and tested for sarcopenia. Please select the statement which best reflects your opinion:

- I would prefer the consultation to be less than 30 minutes
- I would be happy with a consultation length of 30-60 minutes
- I would be happy with a consultation length of greater than 60 minutes, or as long as it takes
- I do not have an opinion on this

Please comment if you wish __________________________________________________

1. The following refers to your opinions on the out-of-pocket costs of a consultation when being assessed for sarcopenia. Please select the statement which best reflects your opinion (select all that apply):

- An out-of-pocket cost for a consultation would be a barrier for me
- An out-of-pocket cost for a consultation WOULD NOT be a barrier for me
- I would be more likely to attend a consultation if there was an out-of-pocket cost
- I would be more likely to attend a consultation if there WAS NO out-of-pocket costs
- I do not have an opinion on this

Please comment if you wish __________________________________________________

**Sarcopenia prevention and treatment**

The following questions ask you to think about what your preferences would be to prevent sarcopenia, or to manage sarcopenia if you were to be diagnosed or have already been diagnosed with sarcopenia.

1. To help prevent sarcopenia (before it has occurred), or manage sarcopenia (when it is present) the type of exercises or action I am/would be willing to consider undertaking include (select all that apply):

- Resistance exercise (this involves exercise with weights or with your own bodyweight, usually in a gym but can be done at home if properly set up)
- Aerobic exercise (this involves exercise that makes you huff and puff, such as running, cycling or walking quickly)
- Aquatic exercises (such as water aerobics)
- Individual exercise classes (with just you and a trainer)
- Group exercise classes (with other people)
- Sports (e.g., lawn bowls, tennis, golf etc)
- Tai Chi
- Yoga
- Dietary changes (such as changing what you eat and drink on the advice of a healthcare professional such as dietitian)
- Dietary changes (including adding healthy things, changing to healthier options, or adding supplements such as protein to your existing diet)
- Prescription medications

*Other exercises or activities I would prefer to undertake include______________*

Please comment if you wish __________________________________________________

1. Please select the one exercise or action you would be most willing to undertake to reduce the chance of getting sarcopenia:

- Resistance exercise (this involves exercise with weights or with your own bodyweight, usually in a gym but can be done at home if properly set up)
- Aerobic exercise (this involves exercise that makes you huff and puff, such as running, cycling or walking quickly)
- Aquatic exercises (such as water aerobics)
- Individual exercise classes (with just you and a trainer)
- Group exercise classes (with other people)
- Sports (e.g., lawn bowls, tennis, golf etc)
- Tai Chi
- Yoga
- Dietary changes (such as changing what you eat and drink on the advice of a healthcare professional such as dietitian)
- Dietary changes (including adding healthy things, changing to healthier options, or adding supplements such as protein to your existing diet)
- Prescription medications

*The exercise or activity I would most prefer to undertake is______________*

Please comment if you wish __________________________________________________

1. How many times a week would you be willing to exercise or participate in activities to reduce your **chances of getting** sarcopenia? Please select the option which best applies to you:

- I do not wish to undertake exercise
- I would be willing to exercise once per week
- I would be willing to exercise two or three times per week
- I would be willing to exercise four or five times per week
- I would be willing to exercise as much as it takes
- I would be willing to exercise as recommended by my health professional.

Please comment if you wish __________________________________________________

1. How many times a week would you be willing to exercise or participate in activities to **help treat** sarcopenia? Please select the option which best applies to you:

- I do not wish to undertake exercise
- I would be willing to exercise once per week
- I would be willing to exercise two or three times per week
- I would be willing to exercise four or five times per week
- I would be willing to exercise as much as it takes
- I would be willing to exercise as recommended by my health professional.

Please comment if you wish __________________________________________________

If I was to undertake exercise training for sarcopenia, I would prefer to do it:

Under the supervision of a health professional at home

Under the supervision of a health professional in a gym

Alone at home

Alone in a gym

With a group at a gym under the supervision of health professional

I don’t mind whether it is at home alone or in a gym, alone or in a group

Please comment if you wish __________________________________________________

The following question refers to how frequently you would prefer to undertake a consultation for sarcopenia with your healthcare professional if you were to be diagnosed.

A consultation would determine whether you still have sarcopenia and whether it has worsened or improved.

A consultation would include a review of your medical history and physical assessments (a measure of strength or something physical) such as measuring muscle strength and walking speed. It could also involve tests (involving a machine), such as a DXA, a type of Xray.

1. Please select the option which best applies to you:

- I would be willing to undertake a sarcopenia consultation every six months or less
- I would be willing to undertake a sarcopenia consultation yearly
- I would be willing to undertake a sarcopenia consultation every second year
- I would be willing to undertake a sarcopenia consultation only when I feel it was necessary
- I would not be willing to undertake further sarcopenia consultations once diagnosed
- I would be willing to undertake sarcopenia consultations as frequently as my health professional recommends

Please comment if you wish __________________________________________________

**Research**

This is a general question to determine individuals’ willingness to participate in different types of research trials exploring treatments if they have, or were to be diagnosed, with sarcopenia. You will not be contacted by the coordinators of this study to participate in any future research studies regardless of your answer to this question.

1. Select all that apply:

- I would be willing to consider being involved in research involving exercise recommendations
- I would be willing to consider being involved in research involving dietary recommendations
- I would be willing to consider being involved in research involving trials of new medications for sarcopenia
- I would not like to be involved in research studies on sarcopenia

Please comment if you wish __________________________________________________

1. Are there any other comments you have, or questions you would like to ask?

**_________________________________________________________________________**
