# Supplementary figures and images for: The Australian and New Zealand Society for Sarcopenia and Frailty Research (ANZSSFR) sarcopenia diagnosis and management task force: Findings from the consumer expert Delphi process
Source: Australas J Ageing. 2022 Dec 8;42(1):251–7. doi: 10.1111/ajag.13164 (PMC10947359; doi:10.1111/ajag.13164)

**Appendix iii – Phase 2 Consumer results infographic**

**
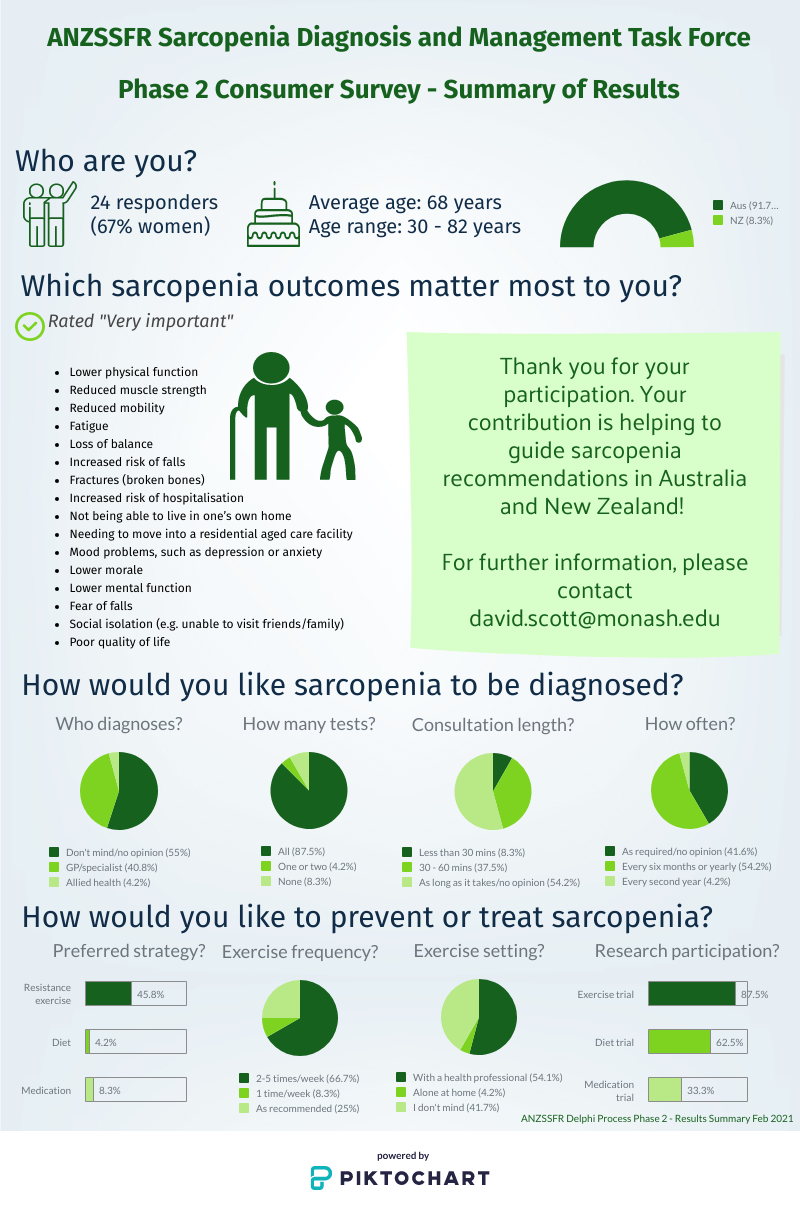
**

Supplement: Supplementary file 3 — Appendix S3 [file AJAG-42-251-s002.docx]
