# Supplementary material for: The Australian and New Zealand Society for Sarcopenia and Frailty Research (ANZSSFR) sarcopenia diagnosis and management task force: Findings from the consumer expert Delphi process
Source: Australas J Ageing. 2022 Dec 8;42(1):251–7. doi: 10.1111/ajag.13164 (PMC10947359; doi:10.1111/ajag.13164)
Supplement: Supplementary file 4 — Appendix S4 [file AJAG-42-251-s003.docx]

**Appendix 4 – Consumer scenarios, questions and preferences**

| **Scenario** | **Preference** | **N (%)** |
| --- | --- | --- |
| *Assessment* |  |  |
| Who do you prefer to diagnose sarcopenia? | I don’t mind who diagnoses sarcopenia | 11 (46) |
|  | I would prefer that my GP diagnose sarcopenia | 5 (20) |
|  | I would prefer that a specialist (e.g., geriatrician) diagnose sarcopenia | 5 (20) |
|  | I do not have an opinion on this | 2 (8) |
|  | I would prefer that an allied health professional (such as a physiotherapist, exercise physiologist, chiropractor, osteopath or dietitian) diagnose sarcopenia | 1 (4) |
| What assessments and tests would you undergo? | I would be willing to undertake all the necessary assessments and tests to diagnose sarcopenia | 21 (88) |
|  | I would prefer not to undertake assessments or tests | 2 (8) |
|  | I would only be willing to undertake one or two assessments to diagnose sarcopenia | 1 (4) |
|  | I would only be willing to undertake one or two tests to diagnose sarcopenia | 0 (0) |
|  | I would prefer to not undertake tests, but would be happy with assessments | 0 (0) |
|  | I do not have an opinion on this | 0 (0) |
| What is your preferred consultation length? | I would be happy with a consultation length of greater than 60 minutes, or as long as it takes | 10 (42) |
|  | I would be happy with a consultation length of 30-60 minutes | 9 (38) |
|  | I do not have an opinion on this | 3 (12) |
|  | I would prefer the consultation to be less than 30 minutes | 2 (8) |
| How frequently would you be willing to undertake a consultation for sarcopenia? | I would be willing to undertake sarcopenia consultations as frequently as my health professional recommends | 9 (38) |
|  | I would be willing to undertake a sarcopenia consultation every six months or less | 7 (29) |
|  | I would be willing to undertake a sarcopenia consultation yearly | 6 (25) |
|  | I would be willing to undertake a sarcopenia consultation every second year | 1 (4) |
|  | I don’t have an opinion on this | 1 (4) |
|  | I would be willing to undertake a sarcopenia consultation only when I feel it was necessary | 0 |
|  | I would not be willing to undertake further sarcopenia consultations once diagnosed | 0 |
| *Prevention* |  |  |
| To prevent sarcopenia, what activities would you be willing to undertake (select all that apply)? | Resistance exercise (this involves exercise with weights or with your own bodyweight, usually in a gym but can be done at home if properly set up) | 18 (75) |
|  | Prescription medications | 17 (71) |
|  | Dietary changes (such as changing what you eat and drink on the advice of a healthcare professional such as dietitian) | 16 (67) |
|  | Aerobic exercise (this involves exercise that makes you huff and puff, such as running, cycling or walking quickly) | 15 (62) |
|  | Group exercise classes (with other people) | 14 (58) |
|  | Aquatic exercises (such as water aerobics) | 11 (46) |
|  | Dietary changes (including adding healthy things, changing to healthier options, or adding supplements such as protein to your existing diet) | 13 (54) |
|  | Individual exercise classes (with just you and a trainer) | 10 (42) |
|  | Sports (e.g., lawn bowls, tennis, golf etc) | 10 (42) |
|  | Tai Chi | 10 (42) |
|  | Yoga | 10 (42) |
| Which one action would you be willing to undertake to prevent sarcopenia? | Resistance exercise (this involves exercise with weights or with your own bodyweight, usually in a gym but can be done at home if properly set up) | 11 (46) |
|  | Individual exercise classes (with just you and a trainer) | 3 (12) |
|  | Aquatic exercises (such as water aerobics) | 2 (8) |
|  | Group exercise classes (with other people) | 2 (8) |
|  | Prescription medications | 2 (8) |
|  | Aerobic exercise (this involves exercise that makes you huff and puff, such as running, cycling or walking quickly) | 1 (4) |
|  | Dietary changes (including adding healthy things, changing to healthier options, or adding supplements such as protein to your existing diet) | 1 (4) |
|  | Other - dancing | 1 (4) |
| How frequently would you be willing to exercise to prevent sarcopenia? | I would be willing to exercise two or three times per week | 13 (54) |
|  | I would be willing to exercise four or five times per week | 3 (12) |
|  | I would be willing to exercise as much as it takes | 3 (12) |
|  | I would be willing to exercise as recommended by my health professional. | 3 (12) |
|  | I would be willing to exercise once per week | 2 (8) |
|  | I do not wish to undertake exercise | 0 |
| *Treatment* | | |
| How frequently would you be willing to exercise to treat sarcopenia? | I would be willing to exercise two or three times per week | 11 (46) |
|  | I would be willing to exercise as much as it takes | 7 (29) |
|  | I would be willing to exercise as recommended by my health professional. | 3 (12) |
|  | I would be willing to exercise once per week | 2 (8) |
|  | I would be willing to exercise four or five times per week | 1 (4) |
|  | I do not wish to undertake exercise | 0 |
| Where and under what supervision would you prefer to exercise to treat sarcopenia? | I don’t mind whether it is at home alone or in a gym, alone or in a group | 10 (42) |
|  | Under the supervision of a health professional in a gym | 8 (33) |
|  | Under the supervision of a health professional at home | 3 (12) |
|  | With a group at a gym under the supervision of health professional | 2 (8) |
|  | Alone at home | 1 (4) |
|  | Alone in a gym | 0 |
| *Research* | | |
| Regarding consumer research, select all that apply | I would be willing to consider being involved in research involving exercise recommendations | 21 (88) |
|  | I would be willing to consider being involved in research involving dietary recommendations | 15 (62) |
|  | I would be willing to consider being involved in research involving trials of new medications for sarcopenia | 8 (33) |
|  | I would not like to be involved in research studies on sarcopenia | 0 |
|  | I don't have an opinion on this | 0 |

Consumer expert preferences across themes of assessment, prevention, treatment and research.
